# 2-(2-Chloro­pyridin-3-yl)-*N*-ethyl-4-methyl-1,3-oxazole-5-carboxamide

**DOI:** 10.1107/S1600536810045885

**Published:** 2010-11-13

**Authors:** Guiqiu Yang, Jiakuang Liang, Haibo Yu, Bin Li

**Affiliations:** a Shenyang Universtity of Chemical Technology, Shenyang 110142, People’s Republic of China; b Agrochemicals Division, Shenyang Research Institute of Chemical Industry, Shenyang 110021, People’s Republic of China

## Abstract

In the title compound, C_12_H_12_ClN_3_O_2_, the dihedral angle between the aromatic rings is 8.42 (10)°. In the crystal, mol­ecules are linked by N—H⋯O hydrogen bonds, generating *C*(4) chains propagating in [001].

## Related literature

For background to derivatives of oxazolyl carb­oxy­lic acids, see: Takechi *et al.* (2000 ▶); Lechel *et al.* (2009 ▶).
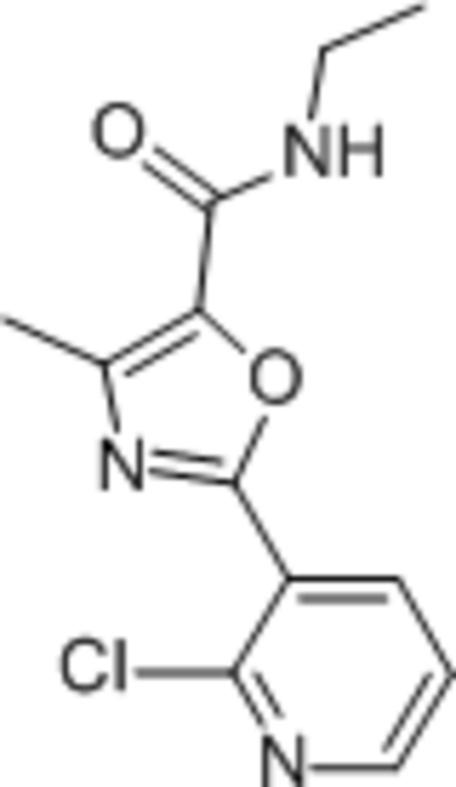


## Experimental

### 

#### Crystal data

C_12_H_12_ClN_3_O_2_
*M* *_r_* = 265.70
Monoclinic, 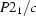

*a* = 8.2143 (12) Å
*b* = 14.545 (2) Å
*c* = 10.4360 (16) Å
β = 97.425 (3)°
*V* = 1236.4 (3) Å^3^
*Z* = 4
Mo *K*α radiation
μ = 0.31 mm^−1^
*T* = 296 K
0.32 × 0.28 × 0.22 mm

#### Data collection

Bruker SMART CCD diffractometer
Absorption correction: multi-scan (*SADABS*; Bruker, 2001 ▶) *T*_min_ = 0.908, *T*_max_ = 0.936
6234 measured reflections
2183 independent reflections
1736 reflections with *I* > 2σ(*I*)
*R*_int_ = 0.027

#### Refinement

*R*[*F* ^2^ > 2σ(*F* ^2^)] = 0.039
*wR*(*F* ^2^) = 0.109
*S* = 1.07
2183 reflections
165 parameters
H-atom parameters constrained
Δρ_max_ = 0.23 e Å^−3^
Δρ_min_ = −0.23 e Å^−3^

### 

Data collection: *SMART* (Bruker, 2001 ▶); cell refinement: *SAINT* (Bruker, 2001 ▶); data reduction: *SAINT*; program(s) used to solve structure: *SHELXS97* (Sheldrick, 2008 ▶); program(s) used to refine structure: *SHELXL97* (Sheldrick, 2008 ▶); molecular graphics: *SHELXTL* (Sheldrick, 2008 ▶); software used to prepare material for publication: *SHELXL97*.

## Supplementary Material

Crystal structure: contains datablocks I, global. DOI: 10.1107/S1600536810045885/hb5697sup1.cif

Structure factors: contains datablocks I. DOI: 10.1107/S1600536810045885/hb5697Isup2.hkl

Additional supplementary materials:  crystallographic information; 3D view; checkCIF report

## Figures and Tables

**Table 1:** Hydrogen-bond geometry (Å, °)

*D*—H⋯*A*	*D*—H	H⋯*A*	*D*⋯*A*	*D*—H⋯*A*
N3—H3⋯O2^i^	0.86	2.29	3.115 (2)	161

Symmetry code: (i) 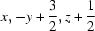.

**Fig. 1.**
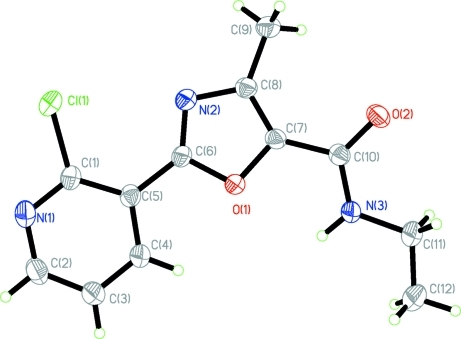
The molecular structure of (I), with atom labels and 30% probability displacement ellipsoids.

**Fig. 2.**
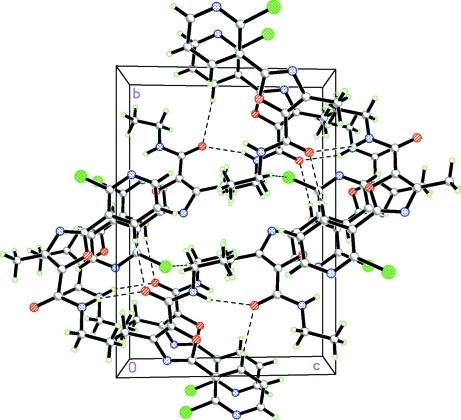
Crystal packing of (I).

## supplementary crystallographic information

### Comment

Derivatives of oxazolyl carboxylic acid are important heterocyclic compounds. They display a broad range of biological, medical and pharmacological properties (Takechi *et al.*, 2000; Lechel *et al.*, 2009). We report the crystal structure of the title compound (I) to determine the structure of the main product in the preparation of derivatives ofoxazolyl carboxylic acid. The molecular structure of (I) (Fig. 1) contains no crystallographically imposed symmetry. The pyridine and oxazole rings in each of the ligands are not coplanar, the dihedral angle formed by the least-squares planes of the benzene and pyrazole rings being equal to 8.8°. Analysis of the crystal packing of (I) shows the existence of N3—H3···O2 interactions, as shown in Fig. 2.

### Experimental

The title compound was synthesized by 2-(2-chloropyridin-3-yl) -4-methyloxazole-5-carbonyl chloride with ethanamine in toluene. The crude products were purified by silica-gel column chromatography and then grown from dichloromethane to afford colorless blocks of (I). To a 100 ml flask ethanamine (0.24 g, 5.40 mmol),triethylamine (0.68 g, 6.75 mmol), 2-(2-chloropyridin-3-yl)-4-methyloxazole-5-carbonyl chloride (1.16 g, 4.50 mmol) and 45 ml toluene were added sequentially. The reaction mixture was reacted for 2 h. After separation through silica gel column chromatography (fluent: ethyl acetate/petroleum ether=1/5), The title compound was gained as a yellow solid (0.42 g, 58%).

Anal. Calcd for C_12_H_12_N_3_: C, 54.25; H, 4.55; N, 15.82. Found: C, 54.33; H, 4.54; N, 15.75. ^1^H NMR(CDCl_3_): 1.27 (t,3*H*, CH_3_), 2.61 (s,3*H*, Ar—CH_3_), 3.50 (m, 2H, CH_2_), 6.29 (br s, 1H, NH), 7.40 (dd, 1H, py—H), 8.42 (dd, 1H, py—H), 8.53 (dd, 1H, py—H).

### Refinement

Although all H atoms were visible in difference maps, they were finally placed in geometrically calculated positions, with C-Hdistances in the range 0.93–0.97Å and N—H distances of 0.86 Å, andincluded in the final refinement in the riding model approximation,with *U*_iso_(H) = 1.2*U*_eq_(C, N) and *U*_iso_(H) = 1.5*U*_eq_(C).

### Crystal data

**Table:**

C_12_H_12_ClN_3_O_2_	*F*(000) = 552
*M**_r_* = 265.70	*D*_x_ = 1.427 Mg m^−^^3^
Monoclinic, *P*2_1_/*c*	Mo *K*α radiation, λ = 0.71073 Å
*a* = 8.2143 (12) Å	Cell parameters from 2185 reflections
*b* = 14.545 (2) Å	θ = 2.4–25.4°
*c* = 10.4360 (16) Å	µ = 0.31 mm^−^^1^
β = 97.425 (3)°	*T* = 296 K
*V* = 1236.4 (3) Å^3^	Block, colorless
*Z* = 4	0.32 × 0.28 × 0.22 mm

### Data collection

**Table:**

Bruker SMART CCD diffractometer	2183 independent reflections
Radiation source: fine-focus sealed tube	1736 reflections with *I* > 2σ(*I*)
graphite	*R*_int_ = 0.027
ω scans	θ_max_ = 25.0°, θ_min_ = 2.4°
Absorption correction: multi-scan (*SADABS*; Bruker, 2001)	*h* = −9→9
*T*_min_ = 0.908, *T*_max_ = 0.936	*k* = −17→17
6234 measured reflections	*l* = −12→8

### Refinement

**Table:**

Refinement on *F*^2^	Primary atom site location: structure-invariant direct methods
Least-squares matrix: full	Secondary atom site location: difference Fourier map
*R*[*F*^2^ > 2σ(*F*^2^)] = 0.039	Hydrogen site location: inferred from neighbouring sites
*wR*(*F*^2^) = 0.109	H-atom parameters constrained
*S* = 1.07	*w* = 1/[σ^2^(*F*_o_^2^) + (0.0583*P*)^2^ + 0.2339*P*] where *P* = (*F*_o_^2^ + 2*F*_c_^2^)/3
2183 reflections	(Δ/σ)_max_ < 0.001
165 parameters	Δρ_max_ = 0.23 e Å^−^^3^
0 restraints	Δρ_min_ = −0.23 e Å^−^^3^

### Special details

**Table:**

Geometry. All esds (except the esd in the dihedral angle between two l.s. planes) are estimated using the full covariance matrix. The cell esds are taken into account individually in the estimation of esds in distances, angles and torsion angles; correlations between esds in cell parameters are only used when they are defined by crystal symmetry. An approximate (isotropic) treatment of cell esds is used for estimating esds involving l.s. planes.
Refinement. Refinement of F^2^ against ALL reflections. The weighted R-factor wR and goodness of fit S are based on F^2^, conventional R-factors R are based on F, with F set to zero for negative F^2^. The threshold expression of F^2^ > 2sigma(F^2^) is used only for calculating R-factors(gt) etc. and is not relevant to the choice of reflections for refinement. R-factors based on F^2^ are statistically about twice as large as those based on F, and R- factors based on ALL data will be even larger.

### Fractional atomic coordinates and isotropic or equivalent isotropic displacement parameters (Å^2^)

**Table:**

	*x*	*y*	*z*	*U*_iso_*/*U*_eq_	
Cl1	0.52313 (8)	1.15459 (4)	0.30039 (6)	0.0650 (2)	
O1	0.81459 (15)	0.89027 (8)	0.35667 (12)	0.0424 (3)	
O2	0.91578 (18)	0.72507 (9)	0.12724 (13)	0.0542 (4)	
N1	0.6093 (2)	1.15962 (11)	0.54565 (19)	0.0582 (5)	
N2	0.6424 (2)	0.96872 (10)	0.21507 (15)	0.0449 (4)	
N3	0.9699 (2)	0.72633 (10)	0.34513 (15)	0.0476 (4)	
H3	0.9523	0.7530	0.4156	0.057*	
C1	0.6202 (2)	1.10823 (12)	0.4431 (2)	0.0457 (5)	
C2	0.6806 (3)	1.12864 (15)	0.6593 (2)	0.0625 (6)	
H2	0.6733	1.1642	0.7324	0.075*	
C3	0.7641 (3)	1.04702 (15)	0.6739 (2)	0.0636 (6)	
H3A	0.8122	1.0278	0.7551	0.076*	
C4	0.7752 (3)	0.99428 (13)	0.56647 (19)	0.0525 (5)	
H4	0.8320	0.9388	0.5744	0.063*	
C5	0.7019 (2)	1.02335 (12)	0.44587 (18)	0.0420 (4)	
C6	0.7133 (2)	0.96435 (12)	0.33294 (18)	0.0399 (4)	
C7	0.8032 (2)	0.84506 (11)	0.23930 (18)	0.0402 (4)	
C8	0.6982 (2)	0.89204 (12)	0.15339 (18)	0.0416 (4)	
C9	0.6388 (3)	0.87207 (15)	0.0153 (2)	0.0567 (6)	
H9A	0.7179	0.8936	−0.0378	0.085*	
H9B	0.5359	0.9027	−0.0091	0.085*	
H9C	0.6242	0.8070	0.0038	0.085*	
C10	0.9008 (2)	0.76014 (12)	0.23233 (18)	0.0412 (4)	
C11	1.0747 (3)	0.64501 (15)	0.3517 (2)	0.0627 (6)	
H11A	1.0109	0.5931	0.3149	0.075*	
H11B	1.1631	0.6554	0.3001	0.075*	
C12	1.1446 (4)	0.6227 (2)	0.4837 (3)	0.0857 (9)	
H12A	1.2037	0.6749	0.5220	0.129*	
H12B	1.2181	0.5715	0.4827	0.129*	
H12C	1.0580	0.6070	0.5333	0.129*	

### Atomic displacement parameters (Å^2^)

**Table:**

	*U*^11^	*U*^22^	*U*^33^	*U*^12^	*U*^13^	*U*^23^
Cl1	0.0845 (4)	0.0471 (3)	0.0613 (4)	0.0194 (3)	0.0012 (3)	0.0078 (2)
O1	0.0535 (8)	0.0366 (7)	0.0360 (7)	0.0063 (6)	0.0015 (6)	−0.0004 (5)
O2	0.0808 (10)	0.0446 (8)	0.0379 (8)	0.0047 (7)	0.0104 (7)	−0.0043 (6)
N1	0.0718 (12)	0.0405 (9)	0.0626 (12)	0.0066 (8)	0.0098 (10)	−0.0062 (8)
N2	0.0531 (10)	0.0376 (8)	0.0428 (9)	0.0036 (7)	0.0016 (8)	0.0025 (7)
N3	0.0633 (10)	0.0408 (9)	0.0387 (9)	0.0139 (7)	0.0071 (8)	0.0002 (7)
C1	0.0502 (11)	0.0347 (10)	0.0524 (12)	0.0005 (8)	0.0078 (9)	0.0015 (8)
C2	0.0843 (16)	0.0496 (12)	0.0541 (14)	0.0016 (12)	0.0106 (12)	−0.0126 (11)
C3	0.0910 (17)	0.0517 (13)	0.0461 (13)	0.0102 (12)	0.0011 (12)	−0.0032 (10)
C4	0.0688 (14)	0.0396 (10)	0.0481 (12)	0.0090 (9)	0.0034 (10)	−0.0018 (9)
C5	0.0474 (11)	0.0339 (9)	0.0453 (11)	−0.0019 (8)	0.0080 (9)	0.0023 (8)
C6	0.0454 (10)	0.0308 (9)	0.0431 (11)	0.0008 (7)	0.0048 (9)	0.0039 (7)
C7	0.0502 (11)	0.0345 (9)	0.0353 (10)	−0.0022 (8)	0.0029 (8)	−0.0011 (7)
C8	0.0479 (11)	0.0375 (10)	0.0387 (10)	−0.0033 (8)	0.0031 (8)	0.0016 (8)
C9	0.0672 (14)	0.0549 (12)	0.0442 (12)	0.0043 (11)	−0.0069 (10)	−0.0020 (10)
C10	0.0500 (11)	0.0348 (9)	0.0392 (10)	−0.0035 (8)	0.0072 (9)	−0.0006 (8)
C11	0.0796 (16)	0.0500 (13)	0.0578 (14)	0.0237 (11)	0.0068 (12)	0.0004 (10)
C12	0.103 (2)	0.0863 (18)	0.0661 (17)	0.0496 (16)	0.0029 (15)	0.0079 (14)

### Geometric parameters (Å, °)

**Table:**

Cl1—C1	1.733 (2)	C4—C5	1.389 (3)
O1—C6	1.364 (2)	C4—H4	0.9300
O1—C7	1.383 (2)	C5—C6	1.471 (3)
O2—C10	1.230 (2)	C7—C8	1.347 (2)
N1—C1	1.318 (3)	C7—C10	1.479 (3)
N1—C2	1.332 (3)	C8—C9	1.489 (3)
N2—C6	1.293 (2)	C9—H9A	0.9600
N2—C8	1.395 (2)	C9—H9B	0.9600
N3—C10	1.333 (2)	C9—H9C	0.9600
N3—C11	1.459 (2)	C11—C12	1.459 (3)
N3—H3	0.8600	C11—H11A	0.9700
C1—C5	1.404 (3)	C11—H11B	0.9700
C2—C3	1.370 (3)	C12—H12A	0.9600
C2—H2	0.9300	C12—H12B	0.9600
C3—C4	1.372 (3)	C12—H12C	0.9600
C3—H3A	0.9300		
			
C6—O1—C7	104.17 (14)	O1—C7—C10	117.74 (15)
C1—N1—C2	117.59 (18)	C7—C8—N2	108.60 (16)
C6—N2—C8	105.37 (15)	C7—C8—C9	130.30 (18)
C10—N3—C11	121.45 (17)	N2—C8—C9	121.08 (17)
C10—N3—H3	119.3	C8—C9—H9A	109.5
C11—N3—H3	119.3	C8—C9—H9B	109.5
N1—C1—C5	124.39 (19)	H9A—C9—H9B	109.5
N1—C1—Cl1	113.91 (15)	C8—C9—H9C	109.5
C5—C1—Cl1	121.70 (15)	H9A—C9—H9C	109.5
N1—C2—C3	123.3 (2)	H9B—C9—H9C	109.5
N1—C2—H2	118.4	O2—C10—N3	123.63 (17)
C3—C2—H2	118.4	O2—C10—C7	120.50 (17)
C2—C3—C4	118.6 (2)	N3—C10—C7	115.86 (16)
C2—C3—H3A	120.7	C12—C11—N3	112.48 (19)
C4—C3—H3A	120.7	C12—C11—H11A	109.1
C3—C4—C5	120.25 (19)	N3—C11—H11A	109.1
C3—C4—H4	119.9	C12—C11—H11B	109.1
C5—C4—H4	119.9	N3—C11—H11B	109.1
C4—C5—C1	115.87 (18)	H11A—C11—H11B	107.8
C4—C5—C6	118.92 (16)	C11—C12—H12A	109.5
C1—C5—C6	125.21 (17)	C11—C12—H12B	109.5
N2—C6—O1	113.63 (16)	H12A—C12—H12B	109.5
N2—C6—C5	131.78 (17)	C11—C12—H12C	109.5
O1—C6—C5	114.57 (16)	H12A—C12—H12C	109.5
C8—C7—O1	108.22 (15)	H12B—C12—H12C	109.5
C8—C7—C10	134.03 (17)		
			
C2—N1—C1—C5	−0.1 (3)	C4—C5—C6—O1	−7.8 (3)
C2—N1—C1—Cl1	179.87 (17)	C1—C5—C6—O1	172.89 (16)
C1—N1—C2—C3	0.3 (4)	C6—O1—C7—C8	−0.41 (19)
N1—C2—C3—C4	0.0 (4)	C6—O1—C7—C10	179.86 (15)
C2—C3—C4—C5	−0.5 (4)	O1—C7—C8—N2	0.6 (2)
C3—C4—C5—C1	0.6 (3)	C10—C7—C8—N2	−179.76 (19)
C3—C4—C5—C6	−178.8 (2)	O1—C7—C8—C9	−178.03 (19)
N1—C1—C5—C4	−0.3 (3)	C10—C7—C8—C9	1.6 (4)
Cl1—C1—C5—C4	179.72 (15)	C6—N2—C8—C7	−0.5 (2)
N1—C1—C5—C6	179.00 (19)	C6—N2—C8—C9	178.25 (18)
Cl1—C1—C5—C6	−1.0 (3)	C11—N3—C10—O2	1.9 (3)
C8—N2—C6—O1	0.2 (2)	C11—N3—C10—C7	−177.52 (18)
C8—N2—C6—C5	−178.29 (19)	C8—C7—C10—O2	11.7 (3)
C7—O1—C6—N2	0.1 (2)	O1—C7—C10—O2	−168.64 (17)
C7—O1—C6—C5	178.89 (15)	C8—C7—C10—N3	−168.8 (2)
C4—C5—C6—N2	170.7 (2)	O1—C7—C10—N3	10.8 (2)
C1—C5—C6—N2	−8.6 (3)	C10—N3—C11—C12	176.9 (2)

### Hydrogen-bond geometry (Å, °)

**Table:**

*D*—H···*A*	*D*—H	H···*A*	*D*···*A*	*D*—H···*A*
N3—H3···O2^i^	0.86	2.29	3.115 (2)	161

Symmetry codes: (i) *x*, −*y*+3/2, *z*+1/2.
